# Case Report: Autoimmune encephalitis and other neurological syndromes with rare neuronal surface antibody in children after hematopoietic stem cell transplantation

**DOI:** 10.3389/fimmu.2023.1274420

**Published:** 2023-10-26

**Authors:** Ming-min Zhang, Jing Wang, Dan Sun, Jing-xuan Wang, Jun-hong Zhang, Jia-wei Xu

**Affiliations:** ^1^ Department of Pediatrics, Union Hospital, Tongji Medical College, Huazhong University of Science and Technology, Wuhan, China; ^2^ Division of Neurology, Wuhan Children’s Hospital, Tongji Medical College, Huazhong University of Science and Technology, Wuhan, China; ^3^ Wisdom Lake Academy of Pharmacy, Xi’an Jiaotong-Liverpool University, Suzhou, China; ^4^ Department of Pediatrics, The Central Hospital of Jingmen, Jingmen, China

**Keywords:** metabotropic glutamate receptor 5, autoimmune encephalitis, hematopoietic stem cell transplantation, epilepsy, graft-versus-host disease

## Abstract

**Introduction:**

Neuronal surface antibody syndromes (NSAS) encompass a growing set of autoimmune neurological disorders, with their predominant clinical presentation being autoimmune encephalitis (AE). The most extensively documented form within NSAS is anti-N-methyl-D-aspartate receptor (NMDAR) autoimmunity. In contrast, other NSAS, such as anti-metabotropic glutamate receptor-5 (mGluR5) autoimmunity, are less common and less comprehensively characterized, particularly in pediatric cases.

**Case description:**

In this instance, we present the case of a 7-year-old girl who exhibited abnormal behaviors following hematopoietic stem cell transplantation (HSCT). She received a diagnosis of anti-mGluR5 AE, and her Electroencephalogram (EEG) displayed an increased number of generalized slow waves during wakefulness. Treatment involved intravenous administration of gamma globulin and methylprednisolone, followed by oral prednisone tablets. Levetiracetam was introduced as an antiepileptic therapy during the pulse steroid therapy. Notably, the abnormal behaviors exhibited significant improvement after treatment.

**Conclusions:**

To the best of our knowledge, this is the first report of rare pediatric NSAS involving anti-mGluR5 AE following HSCT. Enhancing our understanding and characterization of this condition may facilitate its recognition and treatment in children. Serum antibody testing could enable early identification and treatment of anti-mGluR5 AE.

## 1 Introduction

AE is a disease triggered by an abnormal autoimmune response in the central nervous system’s neurons. It is characterized by neuropsychiatric symptoms and seizures. The most common types of AE include anti-NMDAR encephalitis, anti-leucine-rich glioma-inactivating-1 protein encephalitis, and anti-contactin protein-related protein-2 encephalitis (1). In 2011, Lancaster et al. reported the presence of mGluR5 antibodies in the serum and cerebrospinal fluid of two patients with Hodgkin lymphoma and limbic encephalitis (2), thereby raising awareness about anti-mGluR5 AE. In children, AE mediated by anti-mGluR5 is exceedingly uncommon, with merely five documented cases reported since the identification of anti-mGluR5 auto-antibodies in 2011 (3, 4). This report presents the first documented case of the diagnosis and treatment of anti-mGluR5 AE in a child following HSCT. This case serves to enhance clinical awareness of central nervous system complications following HSCT and offers valuable insights into the early diagnosis and treatment of anti-mGluR5 AE.

## 2 Case presentation

**Figure 1:**
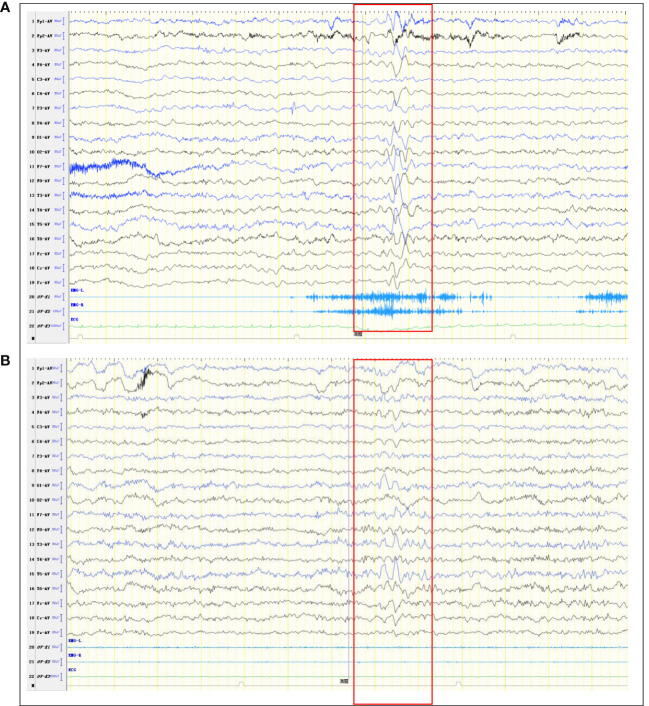
Abnormal EEG. **(A)** Pre-treatment EEG shows More extensive slow waves. **(B)** Post-treatment EEG shows slow waves in the left temporal region (limited reduction than pre-treatment slow waves).

**Figure 2:**
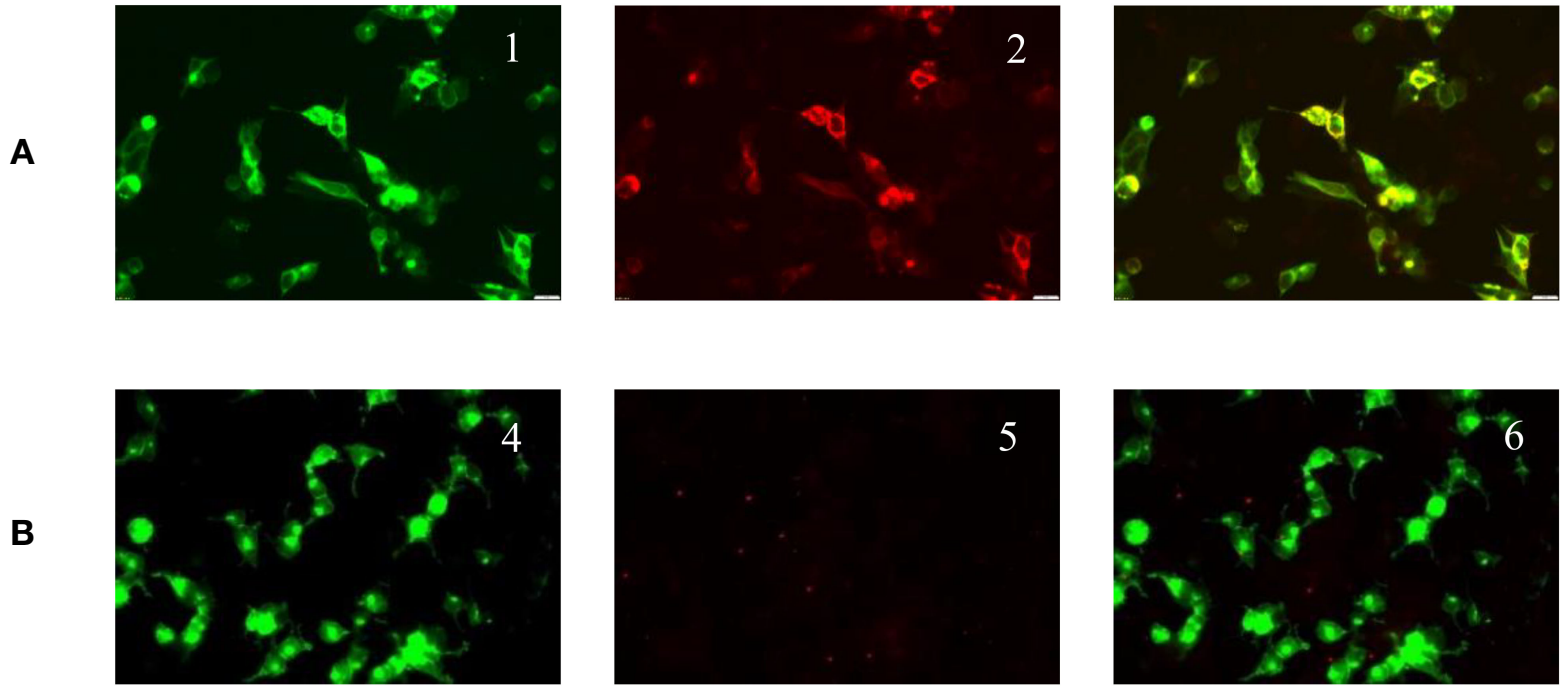
Positive serum anti-mGluR5 IgG expression (double immunofluorescence cell staining; original magnification 400×). **(A)** Serum tested. mGluR5 high expression plasmid transfected cells show green fluorescence (1). Anti-mGluR5 antibodies show red fluorescence (2). mGluR5 antigen antibody fluorescence overlap (3). **(B)** Negative control. GFP-transfected cells (4). Negative mGluR5 antibodies immunofluorescence (5). Fluorescence overlap (6).

On March 14, 2022, a 7-year-old girl was admitted to Wuhan Children's Hospital, Tongji Medical College, Huazhong University of Science and Technology due to noticeable behavioral changes occurring over the past months. These changes included episodes of eye rolling, mouth twitching, and pronounced mouth breathing, lasting approximately 3-5 minutes, particularly when fatigued, happening about 4-5 times per day. Shortly after these episodes, the child would suddenly rise and move about in a seated position, engaging in self-talk and hand-and-foot movements for several hours. The child had been diagnosed with epilepsy at the age of 1, effectively managed with oral levetiracetam. In March 2021, the patient developed intermittent fever and a decrease in blood cell counts. After a thorough examination, the patient was diagnosed with Shwachman-Diamond syndrome (SDS), a genetic condition marked by bone marrow failure and an elevated risk of hematological malignancies. Using whole-exome sequencing, we identified a homozygous splice site variant and this c.258 + 2T>C variant at the 5*’* splice site (ss) is associated with aberrant pre-mRNA splicing due to the usage of an upstream cryptic 5*’*ss at positions c.251-252, eventually resulting in an 8-bp deletion and frameshift (84Cfs3) (5). In November 2021, the patient underwent HSCT from an unrelated HLA9/10-compatible donor at the Department of Pediatrics, Union Hospital Affiliated to Tongji Medical College, Huazhong University of Science and Technology. There were no signs of acute or chronic graft-versus-host disease after HSCT. There was no significant personal history of trauma, infections, tuberculosis exposure, toxin exposure, allergies, or familial metabolic diseases. Upon admission, the patient exhibited stable vital signs, appropriate responsiveness, and normal brain function. Physical examinations revealed a supple neck with non-palpable superficial lymph nodes. Assessments of the heart, lungs, abdomen, and limb muscle strength were normal. Kernig’s and Brudzinski signs were negative, and the heel-knee-shin test showed stability. Imaging tests, including diffusion-weighted magnetic resonance imaging (MRI) of the head, computed tomography scans of the chest, abdomen, and pelvis, superficial lymph node ultrasound, and positron emission tomography-computed tomography, showed no significant abnormalities. The EEG indicated a dominant rhythm without prominence, increased slow wave activity during wakefulness, and occasional paroxysmal multi-spike slow waves during sleep (**Figure 1A**). Integrated sensory and cognitive assessments reported mild proprioceptive and body coordination issues. The self-rating anxiety scale, self-rating depression scale, children’s psychological counseling test report, and China-Wechsler children’s intelligence scale all exhibited normal results. Laboratory tests, including blood counts, blood coagulation, urine and stool routines, liver and kidney function, thyroid function, C-reactive protein, procalcitonin, electrolyte levels, myocardial enzymes, blood ammonia, lactic acid, blood glucose, anti-nuclear antibodies, and anti-extractable nuclear antigen antibodies, all returned within normal ranges. Lymphocyte subsets detection displayed abnormalities (**Table 1**). The patient tested positive for Epstein-Barr virus core antigen, Epstein-Barr virus capsid antigen, cytomegalovirus antibodies, and herpes simplex virus I and II immunoglobulin G (IgG). However, fungal glucan, galactomannan tests, pre-transfusion infectious disease screening, tuberculosis microarray, and parvovirus B-19 yielded negative results. Routine cerebrospinal fluid biochemistry, exfoliative cytology, bacterial cultures, and smears (for common bacteria, cryptococci, and fungi) were all unremarkable. Moreover, 12 cerebrospinal fluid AE antibodies, along with oligoclonal bands and myelin basic protein, tested negative (**Table 1**). Bone marrow cytology and immunophenotypes were generally normal. Double immunofluorescence cell staining revealed elevated anti-mGluR5 antibody levels in the serum (1:1000) (**Figure 2**). A final diagnosis of anti-mGluR5 AE was established. The patient received treatment with intravenous gamma globulin (400 mg/kg daily for 5 consecutive days) and methylprednisolone (20 mg/kg daily for 5 consecutive days), followed by oral prednisone. During hormonal shock therapy, omeprazole was administered for gastric protection, along with calcium and potassium supplementation. Levetiracetam and trihexyphenidyl were concurrently prescribed for epilepsy and dystonia, respectively. The abnormal behaviors notably improved after treatment, and EEG results improved (**Figure 1B**). A re-examination in January 2023 revealed the absence of an SBDS gene mutation in peripheral blood. The patient remained symptom-free at the last follow-up in August 2023.

**Table 1:** Summary of diagnostic evaluation.

Test	Value	Normal range
Natural killer cell (%)	2.53	6.9-19.3
CD19+ B-lymphocyte (%)	7.78	13.4-23.3
CD3+CD4+ T-lymphocyte (%)	13.43	27.8-44.1
CD3+CD8+ T-lymphocyte (%)	64.76	18.2-30.6
CD4+/CD8+	0.21	1.03-2.09
Anti-nuclear antibody	Negative	<1:80
Extractable nuclear antigens (Jo-1, Ro, La, RNP, Sm)	Negative	
Anti-NMDAR IgG	Negative	
Anti-AMPAR1 IgG	Negative	
Anti-AMPAR2 IgG	Negative	
Anti-LGI1 IgG	Negative	
Anti-C ASPR2 IgG	Negative	
Anti-G ABABR IgG	Negative	
Anti-DPPX IgG	Negative	
Anti-Ig LON5 IgG	Negative	
Anti-Gly Rα1 IgG	Negative	
Anti-mGluR5 IgG	1:1000	Negative
Anti-D2R IgG	Negative	
Anti-GAD65 IgG	Negative	
CSF anti-NMDAR IgG	Negative	
CSF anti-AMPAR1 IgG	Negative	
CSF anti-AMPAR2 IgG	Negative	
CSF anti-LGI1 IgG	Negative	
CSF anti-C ASPR2 IgG	Negative	
CSF anti-G ABABR IgG	Negative	
CSF anti-DPPX IgG	Negative	
CSF anti-Ig LON5 IgG	Negative	
CSF anti-Gly Rα1 IgG	Negative	
CSF anti-mGluR5 IgG	Negative	
CSF anti-D2R IgG	Negative	
CSF anti-GAD65 IgG	Negative	
CSF WBC count (10^6^/L)	1	0-10
CSF protein (g/L)	0.18	0.15-0.45
CSF glucose (mmol/L)	3.77	2.2-3.9
CSF oligoclonal bands	0	0–4

CSF, cerebrospinal fluid; NMDAR, N-methyl-D-aspartate receptor; AMPAR1, α-amino-3-hydroxy-5-methyl-4-isoxazole propionic acid type 1 receptor; NMPAR2, α-amino-3-hydroxy-5-methyl-4-isoxazolepropionic acid type 2 receptor; LGI, leucine-rich glioma inactivating 1 protein; CASPR2, contact protein-related protein 2; GABABR, γ-aminobutyric acid type B receptor; DPPX, dihydroxypeptidase-like protein; IgLON5, IgLON family protein 5; GlyR α1, glycine receptor α1 subunit; mGluR5, metabotropic glutamate receptor 5; D2R, dopamine type 2 receptor; GAD65, glutamate decarboxylase 65.

## 3 Discussion

According to previous reports, central nervous system complications occur in 11–65% of HSCT recipients (90% postmortem), accounting for 9–17% of death. These complications encompass infections, tumors, cerebrovascular issues, and autoimmune diseases (6). In this case, the patient displayed sudden behavioral changes with no prior infection history. The patient had a background of congenital bone marrow failure and finished HSCT. We ruled out infections, tumors, cerebrovascular abnormalities, graft-versus-host disease, and genetic metabolic issues. The diagnosis of anti-mGluR5 AE was based on clinical symptoms, positive serum anti-mGluR5 IgG levels, and increased slow waves on EEG. This case expands our understanding of central nervous system complications post-HSCT.

Glutamate serves as a primary excitatory neurotransmitter in the central nervous system, accounting for nearly 50% of intercellular synaptic signaling (7). GluRs play a pivotal role in excitatory synaptic transmission and are associated with mental, neurodevelopmental, and neurodegenerative disorders, including schizophrenia, autism, Parkinson’s, and Huntington’s disease (3). GluRs are classified into glutamate-gated ion channel type receptors and G protein-coupled metabotype receptors (mGluRs). mGluRs are further categorized into three classes based on sequence homology and signaling mechanisms. Class I mGluRs (mGluR1 and mGluR5) activate phospholipase C, triggering intracellular calcium release. Meanwhile, class II (mGluR2 and mGluR3) and class III (mGluR4 and mGluR6–8) mGluRs inhibit adenylyl cyclase (7). Anti-mGluR5 antibodies have been detected in Hodgkin’s lymphoma and limbic encephalitis (including Ophelia syndrome) patients but are rare. These antibodies target hippocampal nerve fibers and neuron surface-related antigens in rats, leading to increased synaptic glutamate levels. Excessive glutamate can harm the brain through excitotoxicity, complement fixation, apoptosis, and induce seizures, ataxia, and behavioral and cognitive abnormalities (8, 9). Elevated glutamic acid concentrations can lead to intracellular calcium overload, protease activation, and cellular damage (10).

Autoimmune diseases, such as cytopenia, thyroid dysfunction, and myasthenia gravis, are common post-HSCT. Chronic graft-versus-host disease closely mimics other autoimmune disorders like scleroderma, Sjogren’s syndrome, and primary biliary cirrhosis (11). The exact mechanisms of AE after HSCT remain unclear. They could be linked to donor-derived autoimmune lymphocytes or immune dysregulation post-transplantation. Factors contributing to post-transplant autoantibody production are multifaceted and involve genetics, environmental factors, and donor cell characteristics (12). In this case, the patient was still in the immune recovery phase after HSCT, and the imbalance between autoregulatory and autoreactive T lymphocytes led to B cells producing anti-mGluR5 antibodies.

Neuronal autoantibodies are highly prevalent in both adults and children, with positive anti-mGluR5 antibodies often lead to neuropsychiatric symptoms, encephalopathies, movement disorders, and seizures. Until to 2023, only five cases of pediatric anti-mGluR5 AE have been reported in PubMed, Cochrane Library, and CNKI using the search terms “chlidren”, “anti-mGluR5 antibody” and “AE” (3, 4). This report presents the first case of anti-mGluR5 AE following HSCT. Based on previous reports and the current pediatric anti-mGluR5 AE cases, it can be concluded that anti-mGluR5 AE patients may typically exhibit: 1. Prodromal symptoms with headaches; 2. An association with Hodgkin’s lymphoma;3. Main clinical features of limbic encephalitis with neuropsychiatric abnormalities; 4. MRI abnormalities; 5. Increased cell count and oligoclonal bands in cerebrospinal fluid; 6. Responsiveness to immunotherapy and/or tumor treatment; 7. Presence of anti-mGluR5 IgG in cerebrospinal fluid, and occasionally in serum; 8. Recurring neurological symptoms. Details are provided in **Table 2**.

**Table 2:** Clinical characteristics of the six reported pediatric anti-mGluR5 AE cases.

No.	Reference	Gender/Age (y)	Prodrome	Main clinical manifestations	Concurrent tumor	Cerebrospinal fluid biochemistry	Anti-mGluR5 antibody	MRI	Treatment method	Prognosis/relapse (yes or no)
1	Spatola et al. (3)	Male/15	Headache and nausea	Confusion, visual hallucinations, auditory hallucinations, decreased concentration, and status epilepticus	Hodgkin’s lymphoma	Nucleated cells: 114 × 10^6^/L; oligoclonal band (+)	Serum: negative; CSF: +	Restricted diffusion in bilateral occipital cortices	Tumor treatment	Full recovery/no
2	Spatola et al. (3)	Male/16	Headache	Mental disturbances, hallucinations, poor sleep, dystonia, and generalized seizures	Hodgkin’s lymphoma	Nucleated cells: 31 × 10^6^/L; oligoclonal band (+)	Serum: > 1/1280; CSF: 1/20	Normal	Tumor treatment, hormonal therapy, and plasma exchange	Full recovery/yes
3	Spatola et al. (3)	Female/6	Rash, headache, and flu-like symptoms	Status epilepticus, memory loss, dystonia, ataxia, and degeneration of speech motor function	Hodgkin’s lymphoma	Nucleated cells: 21 × 10^6^/L; oligoclonal band (-)	Serum: negative; CSF: 1/10	Bilateral frontal cortex, right occipital cortex, and cerebellar high signals	Hormonal therapy, gamma globulin, and rituximab	Partial recovery/no
4	Spatola et al. (3)	Male/15	None	Facial palsy, abnormal behavior, memory loss, visual hallucinations, and insomnia	Hodgkin’s lymphoma	Nucleated cells: 45 × 10^6^/L; oligoclonal band (+)	Serum: 1/1280; CSF: 1/640	Normal	Tumor treatment, hormonal therapy, and gamma globulin	Partial recovery/no
5	Chen et al. (4)	Female/12	None	Seizures and memory loss	None	Normal nucleated cells; oligoclonal band (+)	Serum: negative; CSF: 1/32	Normal	Hormonal therapy and gamma globulin	Partial recovery/no
6	Present case	Female/7	None	Abnormal behavior and seizures	None	Normal nucleated cells; oligoclonal band (-)	Serum:1/1000; Cerebrospinal fluid: negative	Normal	Hormonal therapy and gamma globulin	Full recovery/no

Immunotherapy may be effective due to the direct pathogenic impact of neuronal surface antibodies. Treatment approaches for anti-mGluR5 AE are based on anti-NMDAR encephalitis and include high-dose intravenous corticosteroids, intravenous immunoglobulins, and plasma exchange. Symptomatic and supportive treatments, including antipsychotic and antiepileptic medications, are administered as needed. If initial treatments prove ineffective or recovery is slow, second-line immunotherapy with rituximab or cyclophosphamide may be considered (13, 14). Maintenance therapy with oral steroid is initiated after controlling acute symptoms and gradually tapered over several months. In cases of disease recurrence, mycophenolate mofetil and azathioprine may be used in combination with oral steroid therapy. Given that most anti-mGluR5 AE cases are triggered by tumors, immunotherapy is typically administered in conjunction with standard tumor treatments. Patients with latent tumors, poor responses to immunotherapy, or relapses after initial improvement should be reevaluated after several months.

## 4 Conclusion

The impairment of glutamatergic synaptic transmission caused by anti-neuronal surface GluR antibodies is now widely acknowledged as a significant factor in AE among humans. Conducting serum antibody tests on HSCT patients, especially those lacking autoimmune antibodies in their cerebrospinal fluid, can facilitate the early detection and treatment of anti-mGluR5 AE.

## Data availability statement

The original contributions presented in the study are included in the article/**Supplementary Material**. Further inquiries can be directed to the corresponding author.

## Ethics statement

The studies involving humans were approved by Medical Ethics Committee of the Union Hospital, Tongji Medical College, Huazhong University of Science and Technology (No.2017IEC70). The studies were conducted in accordance with the local legislation and institutional requirements. Written informed consent for participation in this study was provided by the participants’ legal guardians/next of kin. Written informed consent was obtained from the individual(s), and minor(s)’ legal guardian/next of kin, for the publication of any potentially identifiable images or data included in this article.

## Author contributions

M-MZ: Writing – original draft, Writing – review & editing. JW: Writing – review & editing, Conceptualization, Data curation, Software. J-XW: Investigation, Software, Writing – review & editing. DS: Formal Analysis, Supervision, Validation, Writing – review & editing. J-HZ: Writing – review & editing, Investigation, Methodology. JW-X: Formal Analysis, Validation, Visualization, Writing – review & editing.
