# Supplementary material for: Case Report: Autoimmune encephalitis and other neurological syndromes with rare neuronal surface antibody in children after hematopoietic stem cell transplantation
Source: Front Immunol. 2023 Oct 26;14:1274420. doi: 10.3389/fimmu.2023.1274420 (PMC10637573; doi:10.3389/fimmu.2023.1274420)
Supplement: Supplementary file 1 [file DataSheet_1.docx]

Supplementary Material

# Supplementary Figures and Tables

## Supplementary Figures

| A | 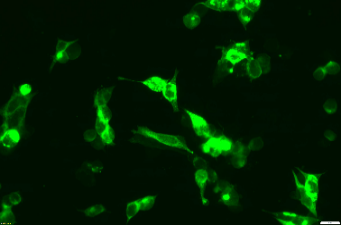 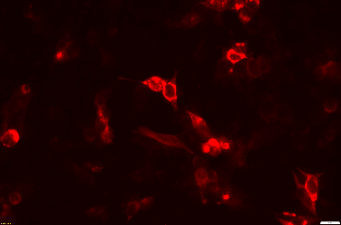 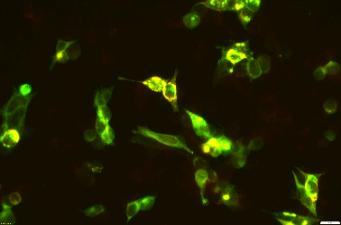  1  2  4  6 |
| --- | --- |
| B | 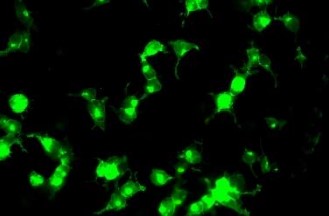 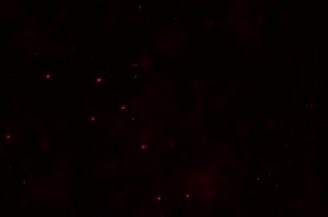 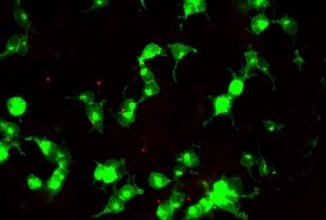  5  6  4 |
| **Supplementary Figure 1.** Positive serum anti-mGluR5 IgG expression (double immunofluorescence cell staining; original magnification 400×). **(A)** Serum tested. mGluR5 high expression plasmid transfected cells show green fluorescence (1). Anti-mGluR5 antibodies show red fluorescence (2). mGluR5 antigen antibody fluorescence overlap (3). **(B)** Negative control. GFP-transfected cells (4). Negative mGluR5 antibodies immunofluorescence（5）. Fluorescence overlap (6). | |

## Supplementary Tables

# Supplementary table 1. Summary of diagnostic evaluation.

| **Test** | **Value** | **Normal range** |
| --- | --- | --- |
| Natural killer cell (%) | 2.53 | 6.9-19.3 |
| CD19+ B-lymphocyte (%) | 7.78 | 13.4-23.3 |
| CD3+CD4+ T-lymphocyte (%) | 13.43 | 27.8-44.1 |
| CD3+CD8+ T-lymphocyte (%) | 64.76 | 18.2-30.6 |
| CD4+/CD8+ | 0.21 | 1.03-2.09 |
| Anti-nuclear antibody | Negative | ＜1：80 |
| Extractable nuclear antigens (Jo-1, Ro, La, RNP, Sm) | Negative |  |
| Anti-NMDAR IgG | Negative |  |
| Anti-AMPAR1 IgG | Negative |  |
| Anti-AMPAR2 IgG | Negative |  |
| Anti-LGI1 IgG | Negative |  |
| Anti-C ASPR2 IgG | Negative |  |
| Anti-G ABABR IgG | Negative |  |
| Anti-DPPX IgG | Negative |  |
| Anti-Ig LON5 IgG | Negative |  |
| Anti-Gly Rα1 IgG | Negative |  |
| Anti-mGluR5 IgG | 1:1000 | Negative |
| Anti-D2R IgG | Negative |  |
| Anti-GAD65 IgG | Negative |  |
| CSF anti-NMDAR IgG | Negative |  |
| CSF anti-AMPAR1 IgG | Negative |  |
| CSF anti-AMPAR2 IgG | Negative |  |
| CSF anti-LGI1 IgG | Negative |  |
| CSF anti-C ASPR2 IgG | Negative |  |
| CSF anti-G ABABR IgG | Negative |  |
| CSF anti-DPPX IgG | Negative |  |
| CSF anti-Ig LON5 IgG | Negative |  |
| CSF anti-Gly Rα1 IgG | Negative |  |
| CSF anti-mGluR5 IgG | Negative |  |
| CSF anti-D2R IgG | Negative |  |
| CSF anti-GAD65 IgG | Negative |  |
| CSF WBC count (10^6^/L) | 1 | 0-10 |
| CSF protein (g/L) | 0.18 | 0.15-0.45 |
| CSF glucose (mmol/L) | 3.77 | 2.2-3.9 |
| CSF oligoclonal bands | 0 | 0–4 |

CSF: cerebrospinal fluid; NMDAR: N-methyl-D-aspartate receptor; AMPAR1: α-amino-3-hydroxy-5-methyl-4-isoxazole propionic acid type 1 receptor; NMPAR2: α-amino-3-hydroxy-5-methyl-4-isoxazolepropionic acid type 2 receptor; LGI: leucine-rich glioma inactivating 1 protein; CASPR2: contact protein-related protein 2; GABABR: γ-aminobutyric acid type B receptor; DPPX: dihydroxypeptidase-like protein; IgLON5: IgLON family protein 5; GlyR α1: glycine receptor α1 subunit; mGluR5: metabotropic glutamate receptor 5; D2R: dopamine type 2 receptor; GAD65: glutamate decarboxylase 65

## Supplementary table 2. Clinical characteristics of the six reported pediatric anti-mGluR5 AE cases.

| **No.** | **Reference** | **Gender/Age (y)** | **Prodrome** | **Main clinical manifestations** | **Concurrent tumor** | **Cerebrospinal fluid biochemistry** | **Anti-mGluR5 antibody** | **MRI** | **Treatment method** | **Prognosis/relapse (yes or no)** |
| --- | --- | --- | --- | --- | --- | --- | --- | --- | --- | --- |
| 1 | Spatola et al.^[5]^ | Male/15 | Headache and nausea | Confusion, visual hallucinations, auditory hallucinations, decreased concentration, and status epilepticus | Hodgkin’s lymphoma | Nucleated cells: 114 × 10^6^/L;  oligoclonal band (+) | Serum: negative;  CSF: + | Restricted diffusion in bilateral occipital cortices | Tumor treatment | Full recovery/no |
| 2 | Spatola et al.^[5]^ | Male/16 | Headache | Mental disturbances, hallucinations, poor sleep, dystonia, and generalized seizures | Hodgkin’s lymphoma | Nucleated cells: 31 × 10^6^/L;  oligoclonal band (+) | Serum: > 1/1280;  CSF: 1/20 | Normal | Tumor treatment, hormonal therapy, and plasma exchange | Full recovery/yes |
| 3 | Spatola et al.^[5]^ | Female/6 | Rash, headache, and flu-like symptoms | Status epilepticus, memory loss, dystonia, ataxia, and degeneration of speech motor function | Hodgkin’s lymphoma | Nucleated cells: 21 × 10^6^/L;  oligoclonal band (-) | Serum: negative;  CSF: 1/10 | Bilateral frontal cortex, right occipital cortex, and cerebellar high signals | Hormonal therapy, gamma globulin, and rituximab | Partial  recovery/no |
| 4 | Spatola et al.^[5]^ | Male/15 | None | Facial palsy, abnormal behavior, memory loss, visual hallucinations, and insomnia | Hodgkin’s lymphoma | Nucleated cells: 45 × 10^6^/L;  oligoclonal band (+) | Serum: 1/1280;  CSF: 1/640 | Normal | Tumor treatment, hormonal therapy, and gamma globulin | Partial recovery/no |
| 5 | Chen  et al.^[11]^ | Female/12 | None | Seizures and memory loss | None | Normal nucleated cells;  oligoclonal band (+) | Serum: negative;  CSF: 1/32 | Normal | Hormonal therapy and gamma globulin | Partial recovery/no |
| 6 | Present case | Female/7 | None | Abnormal behavior and seizures | None | Normal nucleated cells; oligoclonal band (-) | Serum:1/1000;  Cerebrospinal fluid: negative | Normal | Hormonal therapy and gamma globulin | Full recovery/no |
